# The liver injury following ischemia and reperfusion is worse in experimental knockout heterozygote mouse model for expression of connexin 43^1^

**DOI:** 10.1590/s0102-865020190100000003

**Published:** 2019-12-13

**Authors:** Alexandre Maximiliano Trevisan, Bruno Cogliati, Adriana Ribeiro Homem, Thiago Pinheiro Arrais Aloiav, Nelson de Aquino, Jairo Marques Moreira, Leonardo da Cruz Reno, Alexandre Moulin Naumann, Flavio Henrique Ferreira Galvão, Wellington Andraus, Luiz Augusto Carneiro D'Albuquerque

**Affiliations:** I Fellow PhD degree and MSc degree, Postgraduate Program in Medicine Science in Gastroenterology, Department of Gastroenterology, School of Medicine, Medical Investigation Laboratory (LIM 37), Universidade de São Paulo (USP), Brazil. Technical procedures, acquisition of data, statistical analysis, manuscript writing; II PhD, Department of Pathology, School of Veterinary Medicine and Animal Science, USP, Sao Paulo-SP, Brazil. Technical procedures; III PhD, Department of Gastroenterology, School of Medicine, Medical Investigation Laboratory (LIM 37), USP, Sao Paulo-SP, Brazil. Manuscript writing; IV Nursing student, Hospital Albert Einstein, Sao Paulo-SP, Brazil. Technical procedures, acquisition of data; V Fellow Master degree, Postgraduate Program in Medicine Surgical Gastroenterology, School of Medicine, USP, Sao Paulo-SP, Brazil. Statistical analysis, manuscript writing; VI Biologist, Hospital Albert Einstein, Sao Paulo-SP, Brazil. Technical procedures, acquisition of data; VII Fellow Master degree, Postgraduate Program in Medicine Surgical Gastroenterology, School of Medicine, USP, Sao Paulo-SP, Brazil. Technical procedures, acquisition of data, manuscript writing; VIII Assistant Professor, Liver and Gastrointestinal Transplant Division, Department of Gastroenterology, School of Medicine, Coordinator, Medical Investigation Laboratory (LIM 37), USP, Sao Paulo-SP, Brazil. Surgical procedures, manuscript writing, critical revision; USP, Medical Investigation Laboratory (LIM 37), Sao Paulo, SP, Brazil; IX Assistant Professor, Coordinator, Liver and Gastrointestinal Transplant Division, Department of Gastroenterology, School of Medicine, Medical Investigation Laboratory (LIM 37), USP, Sao Paulo-SP, Brazil. Surgical procedures, manuscript writing, critical revision; X Full Professor, Chairman, Liver and Gastrointestinal Transplant Division, Department of Gastroenterology, School of Medicine, Medical Investigation Laboratory (LIM 37), USP, Sao Paulo-SP, Brazil. Conception and design of the study, manuscript writing, critical revision

**Keywords:** GAP junctions, Connexin 43, Ischemia, Reperfusion, Liver, Cell communication, Mice

## Abstract

**Purpose:**

To evaluate that Connexin (Cx43) plays a role in lesions after hepatic ischemia/reperfusion (IR) injury.

**Methods:**

We use Cx43 deficient model (heterozygotes mice) and compared to a wild group. The groups underwent 1 hour ischemia and 24 hours reperfusion. The heterozygote genotype was confirmed by PCR. We analyzed the hepatic enzymes (AST, ALT, GGT) and histology.

**Results:**

The mice with Cx43 deficiency showed an ALT mean value of 4166 ***vs***. 307 in the control group (p<0.001); AST mean value of 7231 ***vs***. 471 in the control group (p<0.001); GGT mean value of 9.4 vs. 1.7 in the control group (p=0.001); histology showed necrosis and inflammation in the knockout group.

**Conclusions:**

This research demonstrated that the deficiency of Cx43 worses the prognosis for liver injury. The topic is a promising target for therapeutics advancements in liver diseases and procedures.

## Introduction

The number of liver surgeries such as transplantation and resection has increased exponentially over recent decades. During surgical processes, it is important to control bleeding through the complete or partial impediment of blood flow to the liver^1^. This procedure leads to oxygen deprivation in the remaining tissue, causing tissue injury^2^.

Despite the restoration of blood flow being essential to prevent irreversible cellular damage, reperfusion itself can aggravate ischemic cellular damage^3^. Liver ischemia, at the same time as minimizing bleeding during surgery, is known to induce hepatocellular stress. Within certain limits, patients are more able to withstand hepatic ischemia than the deleterious effects of large hemorrhages and subsequent blood transfusions and products^4^.

The injury from hepatic ischemia and reperfusion (IR) can be conceived of as a conjunction of circulatory and metabolic alterations that evolve with hepatic dysfunction and tissue damage secondary to the sequential period of the ischemia of the liver (whether at normal temperature or cold) followed by blood reperfusion^5^. The damage arising from IR is one of the main causes of poor functioning of the graft after liver transplantation and directly influences patient survival. In addition, steatotic livers, that are strongly increasing in numbers nowadays, are more susceptible to the IR injury.

Liver injury caused by IR is affected by the ischemia time and by the moment of reperfusion, encompassing a range from biochemical alterations to cellular necrosis^3^ ^,^ ^6^ ^,^ ^7^.

This is a dynamic process resulting from the excessive deposit of extracellular components such as HSCs, CK, cytokines and growth factors that determine an imbalance of homeostatic mechanisms between synthesis and collagen degradation^8^.

Beyond cytokines and other soluble factors promoting the activation and synthesis of collagen by the HSC, they are capable of communicating with one another^9^ and with other cells in the hepatic microenvironment. These cellular interactions are frequently made by gap junctions, in which connexins are responsible for cell-to-cell communication. They are classified according to molecular weight (from 26 to 59 kDa), preceded by Cx, for example: Cx 26, Cx 43, Cx 32. To date, 21 types have been identified^10^.

Connexins have three basic functions: 1 – form hemichannels that allow communication between the cell and the extracellular matrix, 2- form communicating units that allow the transfer of small molecules between adjacent cells and 3 – play an independent role in the activity of channels that control cellular proliferation^11^. Various connexins have tissue-specific expressions, while many tissues have expressions of different connexins.

Connexins can interact with each other, forming homomeric connexons (formed by six identical connexins) or heteromeric (formed by six different connexins). Connexons can also interact with each other, constituting homotypic channels (formed by identical connexons) and heterotypical channels (formed by different connexons). These different combinations result in different functions of the gap junctions formed, because each connexin has different properties of permeability, conductivity, contigency and channel opening^12^.

Connexins are the main protein components of the intercellular junctions found in the liver, and are located in the plasmatic membrane of the cells. Cx32 and Cx26 are expressed in hepatocytes; the first is found uniformly distributed in all regions of the liver, while the second is usually expressed in the periportal acinus^18^ ^,^ ^19^.

The endothelial cells of the hepatic veins and arteries express Cx37 and Cx40, while the majority of non-parenchymal cells express Cx43, including the HSCs^20^, Cks, cholangicytes, oval/progenitor cells and mesothelial cells of the Glisson capsule. Some occasional reports describe the expression of Cx26 in sinusoidal endothelial cells, with extremely low expression of Cx43 or Cx32^20^ (Table 1).

**Table 1:** Connexin expression in different types of hepatic cells

Hepatic Cells	Connexins (Cx)
Hepatocytes	32 and 26
Cholangicytes	43 and 32
Starred cells (Ito)	43 and 26
Kupffer cells	43 and 26
Sinusoidal endothelial cells	26, 43/32
Endothelial cells of vessels and arteries	43
*Mesothelial cells (Glisson capsules)*	43

Source: (modified from Vinken *et al*.^20^).

The expression of Cx43 in the liver can be modified when the organ's cells are stressed^21^. The studies relating to connexin and liver disease do not assess Cx43 in particular, and more specifically in livers that suffer damage during IR. The studies of liver IR are at present restricted to the molecules Cx26 and Cx32. Few studies show the participation of gap junctions in injury during IR. It is known that after the ischemic period, there is remodeling of the hepatic tissue with the participation of cytokines, metalloproteinases, HSCs, Kupffer cells, etc.; however, there are no existing studies of Cx43 in this context.

## Methods

A total of 34 mice, 16 genetically modified, deficient in one of the alleles of Cx43^+/-^, and 18 wild mice C57 BL/6(Cx43^+/+^). All had the genotype PCR for the gene Cx43. All were male, adult (8 weeks), weight +/- 30g.

The animals remained in polycarbonate boxes, in a room with photoperiod of 12 hours of light and 12 hours of dark, under temperature controlled conditions (22 +- 2°C) and an air humidity of 45-60%. They received water (filtered pH +- 7.0) and food (Nuvilab CR1, Nuvital Nutrientes Ltda, Brasil) *ad libitum* for the full experimental period.

All procedures were carried out in compliance with the relevant legislation: Law n. 6638 of 08/05/1979, Law decree 64704 of 17/06/1969, and the Universal Declaration of Animal Rights (Unesco 27/01/1978); all laboratories with biosecurity certificates of FMVZ-USP (CQB n.100/99).

The animals were divided in 3 groups: Sham, Knockout and Wild.

**Sham group** (n=2): underwent laparotomy and sacrificed after 1 hour;

**Knockout group** (n=16): underwent ischemia for 1 hour, and 24 hours reperfusion and then sacrificed;

**Wild group** (n=16): same procedure as the knockout group.

### Genotyping

For the identification and control of the animal genotypes we used a 0.5cm fragment of the tail of each animal. The fragment was digested in an extraction buffer (with proteinase K) for two hours in a water bath at 65°C to extract the DNA. After digestion, a lysis buffer was added, homogenized and the samples were placed in a centrifuge for 20 minutes (15.000 rpm at 4°C). Around 200µl of the aqueous phase were transferred to a new tube, and 400µl of ethanol and 20µl of sodium acetate 3M (pH 5.2) were used in order to extract the DNA. After 10 minutes of centrifuge at 5,000 rpm (4°C), the DNA was washed with 70% ethanol and dissolved in 200µl of TE (Tris/EDTA). After bio photometric quantification, the DNA of each mouse was analyzed by PCR with primers for the Cx43 gene.

Sense 5′CCCCACTCTCACCTATGTCTCC3′

AntiSense 5′ACTTTTGCCGCCTAGCTATCCC3′

The polymerase reaction in each chain (PCR) for Cx43 was carried out in a single cycle, for 2 minutes at 94°C, 35 cycles at 94°C for 30 seconds, 55°C for 1 minute, 72°C for 4 minutes, 1 cycle at 72°C for 4 minutes and 1 cycle at 4°C for 60 minutes.

For the surgical procedure, we developed a protocol from anesthetic induction to post-operative care^23^.

**Figure 1:**
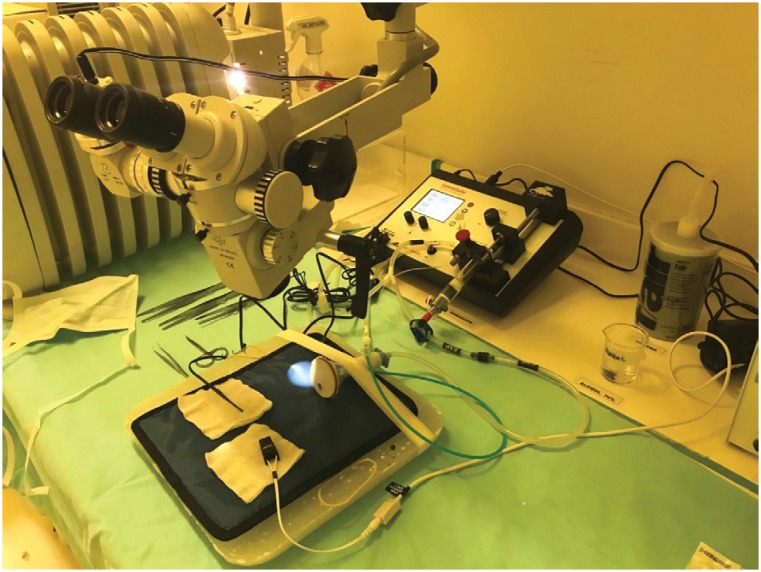
Sommo Suite anesthetic equipment (small animal anesthesia system) of the rodents with a nasal mask and heated bed.

The animals were anesthetized with isoflurane, via inhalator through specific anesthetic equipment for rodents (Fig. 1).

The isoflurane (difluorometil 1-cloro-2,2,2-trifluoretil ether) was used in the pre-anesthetic induction in a percentage of 5% inside a chamber, which was then maintained at 2.5%, which could be increased or decreased according to the procedure time or reaction of the rodents.

**Figure 2:**
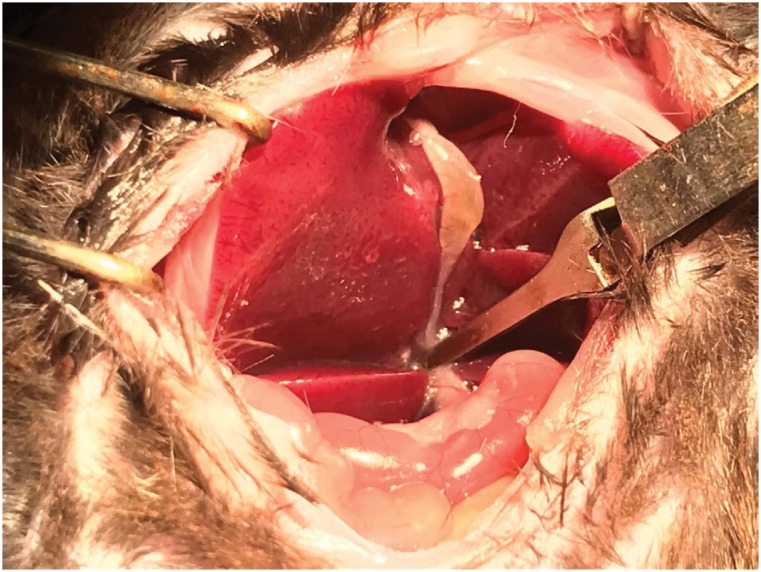
Experimental delineation. Separation via rods exposing the clamp underneath the right lateral lobe.

**Figure 3:**
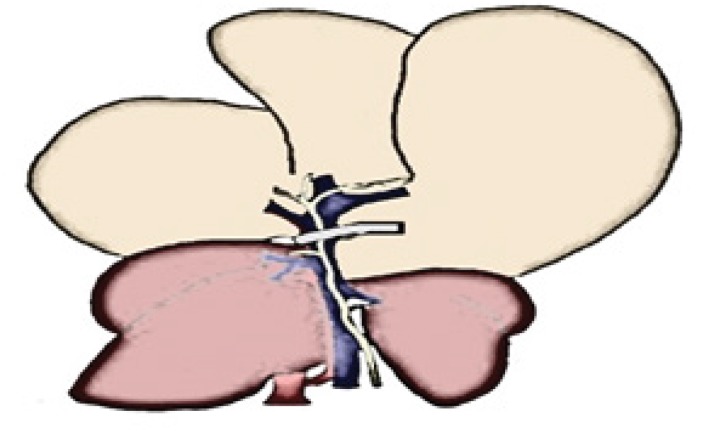
On the left, a diagram of a ventral view of a wild rat's liver prior to clamping. The portal vein is exposed (*blue*), the hepatic artery (*red*) and the common biliary duct (in green). After the triadic clamping, (*the clamp is marked in grey over the portal vein*). Induction of ischemia and color change (*light brown*) of the left lateral lobe and the liver's median lobe.

**Figure 4:**
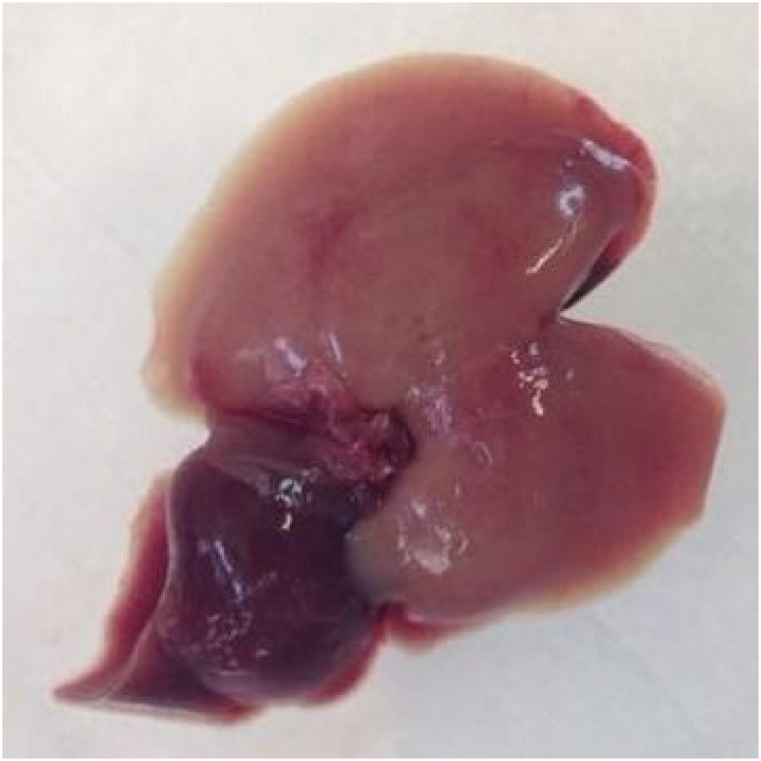
Liver of the wild mouse, after one hour of ischemia (*triad clamping*), observing the color change of the ischemic area (*light red*) of the left lateral lobe and median lobe of the liver (70% of the liver).

A median incision of 3cm in the middle of the abdomen was made up to the xiphoid process. The dissection was made with flexible rods and cotton buds moistened with saline solution. Dissection was continued until exposition of the portal vein and adjacent structures. When adequate exposition was achieved, the pedicle was clamped with a non-traumatic clamp on the exposed structure (portal vein, hepatic artery and biliary duct) immediately below the right lateral lobe (Fig. 2). The medial and left lateral lobes became pale in color (70% of the liver) (Fig. 4), similar to the diagram (Fig. 3). While we waited 60 minutes for ischemia, the rodent's body temperature needed to be maintained. A thermal blanket and heated saline solution in the peritoneum were used. Moistened gauzes were placed on the intestine. After the removal of the clamp, we observed the revascularization of the liver and breathing patterns, before suturing the musculature and skin of the mouse with Mononylon 4-0. Postoperative care involved analgesics, thermic control and inspection of the postoperative wound. All groups were euthanized via isoflurane overdose shortly after reperfusion.

### Biochemical analysis

After anesthesia and before euthanasia, 1ml of blood was collected via a puncture in the abdominal aorta, from both groups (knockout and wild) after 24 hours of reperfusion.

The samples were centrifuged and the following biochemical parameters were analyzed: FA, ALT, AST and GGT. The analyses were made in an automatic biochemical analyzer Vet Test 8008 (QBC Analyzer, IDEXX Laboratories Ltd, Chalfont St Peter, UK).

### Histochemical analysis

The liver tissue removed was fixed with solution of formol (10% formol). This material was placed in 100% ethanol for 12 hours; it was bathed in xilol and prepared for inclusion in paraffin. The slides were stained with Hematoxylin and Picrosirius, and then analyzed via optical microscope by a medical pathologist that did not know the groups to which the tissue samples belonged.

### Statistical analysis

Descriptive analysis: mean, SD, median and percentiles. Non-parametric statistics: two independent groups (Wilcoxon – Mann – Whitney).

## Results

**Figure 5:**
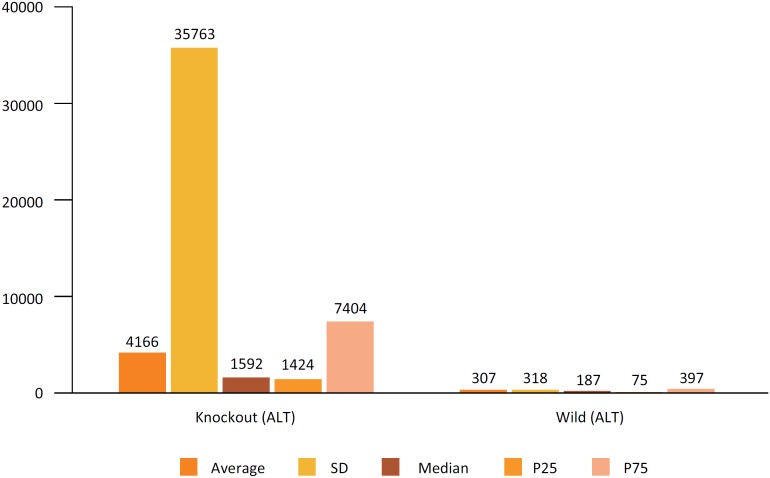
Comparison of hepatic enzyme (ALT) results between the genetically modified group (heterozygous Knockout for Cx43 (GM)) and the control group (wild).

In the evaluation of the hepatic lesion after IR, through the measurement of Alamine transaminase (ALT), the group Knockout heterezigoto, that is, with lower expression of Cx43, showed values significantly higher, when compared with the wild control group (Fig. 5).

**Figure 6:**
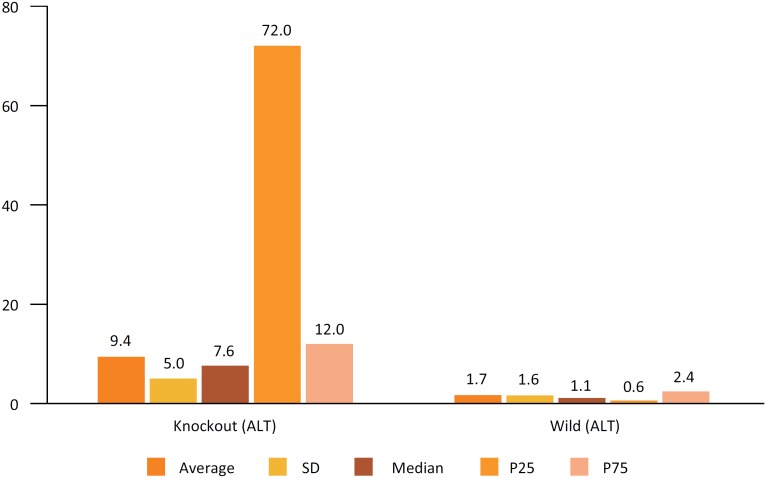
Comparison of hepatic enzyme results (GGT) between the genetically modified group (heterozygous Knockout for Cx43 (GM)) and the control group (wild).

**Figure 7:**
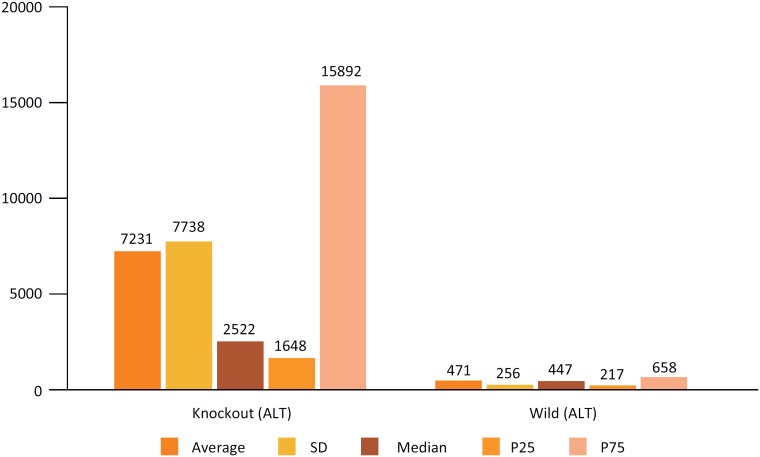
Comparison of liver enzyme results (AST) between the genetically modified group (heterozygous Knockout for Cx43 (GM)) and the control group (wild).

When Aspartate transaminase (AST) was used to assess hepatic IR injury, a difference similar to that found with ALT was observed, with a p near zero (Fig. 6). Also in the GGT evaluation, a significant difference was observed with p = 0.001, confirming that in the group with lower expression of Cx43 the lesion is significantly bigger (Fig. 7).

Animals lacking Cx43 have significantly larger risk of hepatic injury when submitted to this model of liver injury.

**Figure 8:**
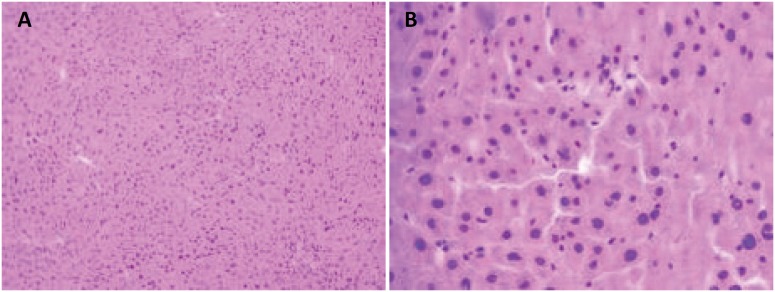
Microscopic appearance of heterozygous Knockout mouse liver for Cx43 subjected to hepatic ischemia for 60 minutes followed by 24 hours of reperfusion. The presence of severe hepatocellular necrosis and sinusoidal inflammatory cells (hematoxylin-eosin. **A**, increase x100, and **B**, increase x400) can be observed.

**Figure 9:**
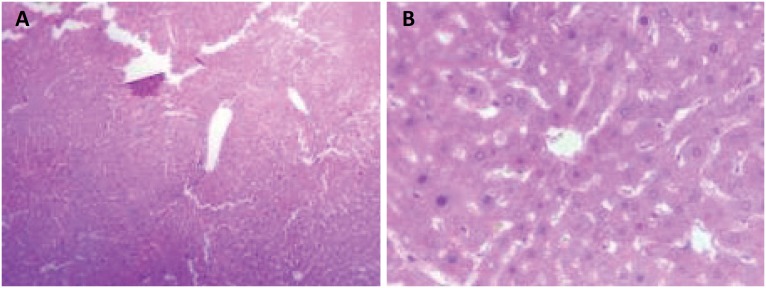
Microscopic examination of wild mouse liver submitted to hepatic ischemia for 60 minutes followed by 24 hours of reperfusion. Discrete parenchitomatous disorganization and vacuolar degeneration may be observed. Absence of hepatocellular necrosis (hematoxylin-eosin. **A**, increase x100, and **B**, increase x400).

In the evaluation 24 hours after the ischemia procedures, the histopathological study demonstrated greater vascular congestion (VC) – sinusoidal, centrolobular and portal space – and hepatocytic necrosis in the knockout animals when compared to the control animals (Figs. 8 and 9).

## Discussion

To our knowledge, this is the first study that demonstrated a worse response to I/R injury in animals with Cx 43 deficiency.

Cx 43 is one of the most studied connexins, perhaps because it appears in so many different types of cell. It was first described by Beyer *et al*.^10^, in rat myocardia. Later, Jhappan *et al*.^28^ described the total longitude of the primary transcript of Cx43 in human and rat myocardia. The gene for Cx43 is located in chromosome 6 (6q22.31; ID: 2697), giving way to a transcript of 14.168 pb, which translates in 382 amino acids. The Cx43 is phosphorylated throughout its lifecycle, suffering conformational alterations, resulting in different isoforms (P0, P1 and P2), presenting distinct properties.

Cx43 channels play important roles mediating the cellular communication between tissues in the vascular, digestive, reproductive and nervous systems^13^. Cx43 is involved in the normal development of the heart. This was initially observed when Cx43 homozygous knockout mice died at birth, due to an obstruction at the exit of the left ventricle^14^ ^,^ ^15^. In the blood vessels, which regulate blood pressure in the vascular system, Cx43 allows the propagation of action potential between cardiomyocytes, together with Cx40 and Cx37. The presence of Cx43 at the mitochondrial level has been described, where it plays an essential protecting role for survival after the ischemia process, associated with the protection it offers in pre-conditioning, which allows the cells to increase their resistance to the ischemia process^16^ ^,^ ^17^. Our study is in accordance with this one, as we found a very big difference between the groups, showing that the Cx 43 has an important role in protecting the liver against injuries.

In 2008, Koch *et al*.^9^ observed that Cx43 is associated with the establishment of functional intercellular communication between active Kupffer cells. The authors show that inflammatory conditions induce Cx43 and activation of CKs in vivo or in vitro that establish functional junctions. Active HSCs express a significant quantity of Cx43 and establish efficient cellular communication, only between them.

Xu *et al*.^29^ showed that the expression of Cx43 in HSCs can be modulated by levels of TGFβ-1 and that the expression of Cx43 interferes directly in the proliferation of these cells. In the same work, using interference RNA for the gene of Cx43, the authors observe that lower expression of Cx43 was associated with a reduction of cellular proliferation of HSC.

Connexins and their channels are frequently found below normal levels in cases of acute liver insufficiency (drug induced), hepatitis, cholestasis, fibrosis, cirrhosis, non-alcoholic steatosis and hepatocellular carcinoma^20^.

There are drugs that can inhibit gap junctions (Cabenoxolone), selectively (TAT-gap 19) and hemichannels^26^. Other circumstances can enhance its expression. Finding the real advantages and disadvantages of cellular communication inhibition may offer new treatment options for the patients.

The experimental model of partial I/R injury showed to be feasible and reproductible using heterozygote animals for Cx 43. Even using a small sample, the changes found were significantly different between the two groups, showing a much worse behavior in the deficiency of Cx 43, proving the importance of conexin in circumstances of liver injury.

## Conclusion

The deficiency of Cx 43 worses the ischemia/reperfusion injury in an experimental model using heterozygote mice for the expression of this conexin.
